# Single-Session Transcatheter Aortic and Tricuspid Valve-in-Valve Implantation in a Patient with Prior Multivalve Surgery and a Chronic Transvenous Pacing Lead: A Case-Based Review of Procedural Strategy

**DOI:** 10.3390/life16091535

**Published:** 2026-09-15

**Authors:** Cristian Mornoș, Laurentiu Pașcalau, Vlad Anton Iliescu, Aniko Mornoș, Daniel-Miron Brie, Horea Feier, Mihai-Andrei Lazăr, Silvius-Alexandru Pescariu, Dragoș Cozma

**Affiliations:** 1 Department VI—Cardiology, “Victor Babes” University of Medicine and Pharmacy of Timisoara, E. Murgu Sq. No. 2, 300041 Timisoara, Romania; mornos.cristian@umft.ro (C.M.); horea.feier@umft.ro (H.F.); pescariu.alexandru@umft.ro (S.-A.P.); dragos.cozma@umft.ro (D.C.); 2 Institute of Cardiovascular Diseases Timisoara, G. Adam Str. No. 13A, 300310 Timisoara, Romania; pascalau_laurentiu@yahoo.com (L.P.); brie_daniel@yahoo.com (D.-M.B.); 3 Research Centre of the Institute of Cardiovascular Diseases Timisoara, G. Adam Str. No. 13A, 300310 Timisoara, Romania; mornosaniko@yahoo.com; 4 Cardio-Thoracic Pathology Department, Carol Davila University of Medicine and Pharmacy, Bulevardul Eroii Sanitari No. 8, 050474 Bucharest, Romania; vladanton.iliescu@gmail.com

**Keywords:** transcatheter aortic valve implantation, tricuspid valve-in-valve, balloon-expandable valve, guidewire pacing, multivalvular heart disease, heart team, lead management

## Abstract

**Background**: The population of patients surviving previous multivalve surgery is expanding, and structural Heart Teams increasingly encounter complex multivalvular disease requiring reintervention, for which redo surgery carries prohibitive risk. Patient selection, sequential procedural planning, prosthesis choice, and, in particular, pacing strategy and protection of pre-existing intracardiac hardware remain unstandardized. **Case Presentation**: We report a 73-year-old woman with a twenty-year history of rheumatic multivalvular heart disease, comprising mechanical mitral valve replacement, tricuspid annuloplasty and pacemaker implantation, redo bioprosthetic tricuspid valve replacement, and critical native aortic stenosis combined with severe bioprosthetic tricuspid regurgitation. After multidisciplinary Heart Team evaluation, the patient underwent successful single-session, sequential transfemoral transcatheter aortic valve implantation followed immediately by tricuspid valve-in-valve implantation using a reversed balloon-expandable valve and a dedicated biventricular guidewire pacing strategy. The sequential interventions achieved immediate hemodynamic success with no lead dysfunction, paravalvular leak, or other acute complications. **Review**: Using this case as a clinical anchor, we present a focused, case-based review of contemporary evidence on single-session double-valve transcatheter intervention. We address patient selection, procedural sequencing, imaging, prosthesis choice, and management of chronic transvenous pacing leads, providing a practical decision-making algorithm to guide Heart Teams in similarly complex scenarios. **Conclusions**: Individualized, multidisciplinary Heart Team planning, including a dedicated biventricular guidewire strategy, can simplify sequential transcatheter treatment of multivalvular disease in patients with pre-existing intracardiac hardware. This case-based review provides a pragmatic, hypothesis-generating framework to support clinical decision-making as the body of evidence for combined double-valve transcatheter interventions continues to evolve.

## 1. Introduction

Advances in cardiac surgery, prosthetic valve technology, and structural heart interventions have profoundly changed the natural history of patients with complex valvular heart disease. As life expectancy after valve surgery continues to increase, structural Heart Teams are increasingly confronted with patients who present years, or even decades, after their original operation with progressive degeneration of surgical repairs or bioprosthetic valves, frequently affecting more than one cardiac valve at a time.

Rheumatic heart disease remains the paradigmatic example of this long multivalvular trajectory. It typically begins with damage to the mitral valve after acute rheumatic fever and, over subsequent decades, often extends to the aortic and tricuspid valves, producing a cumulative burden of valvular, ventricular, and rhythm disease [1]. Although its incidence has fallen substantially in high-income countries, rheumatic heart disease still caused an estimated 373,000 deaths and affected nearly 55 million people worldwide in the most recent Global Burden of Disease (2021) analysis, and structural collaborative assessment continues to encounter its late multivalvular consequences even where acute rheumatic fever itself has become rare [2].

Redo cardiac surgery remains the conventional treatment for failed surgical bioprostheses and for newly developed severe valvular disease, but its risk escalates sharply with clinical deterioration and with each additional sternotomy. In contemporary series of reoperative valve surgery, operative mortality has ranged from as low as 1.4% for elective procedures to approximately 8% for urgent and 37.5% for emergency operations, and mortality has been reported to rise from around 1.6% in patients in New York Heart Association (NYHA) functional class I to 20.8% in NYHA class IV [3]. Advanced age, pulmonary hypertension, chronic atrial fibrillation, permanent pacing systems, hepatorenal dysfunction, and impaired ventricular function further compound this risk, particularly after two or more previous sternotomies [3].

Consequently, transcatheter therapies have progressively expanded beyond isolated aortic valve replacement. Transcatheter tricuspid valve interventions—whether repair or replacement—now carry a Class IIa (Level of Evidence A) recommendation for symptomatic, high-risk patients with severe tricuspid regurgitation in the most recent international guidelines, provided severe right ventricular (RV) dysfunction or precapillary pulmonary hypertension are absent [4]. Valve-in-valve replacement of degenerated tricuspid bioprostheses using balloon-expandable transcatheter platforms has been increasingly reported as a technically feasible and clinically effective alternative to redo surgery: the largest published multicenter registry documented successful implantation in 150 of 152 attempted procedures (98.7%) [5], and subsequent single-center case series have confirmed good early hemodynamic outcomes and low procedural complication rates [6].

Even so, combined treatment of severe native aortic stenosis (AS) and structurally deteriorated tricuspid bioprosthesis during a single procedural session remains uncommon and has so far been described mainly in isolated case reports [7,8]. Cases in which procedural planning must simultaneously address a mechanical mitral prosthesis and a permanent transvenous pacing lead crossing the tricuspid prosthesis are rarer still, and the presence of chronic intracardiac hardware adds a further layer of complexity that is only beginning to receive systematic attention in the interventional and electrophysiology literature [9].

In such complex scenarios, procedural success depends not only on accurate preprocedural imaging and valve selection, but also on careful planning of guidewire positioning, pacing strategy, device exchanges, and protection of pre-existing intracardiac hardware. Minimizing unnecessary catheter manipulation may reduce procedural complexity and the risk of complications associated with repeated instrumentation of previously operated cardiac chambers.

We report the case of a patient with a nearly twenty-year history of rheumatic multivalvular heart disease who underwent single-stage treatment of critical native AS and a structurally degenerated tricuspid bioprosthesis. The distinguishing feature of this intervention was the deliberate use of dedicated stiff guidewires positioned in both ventricles before valve deployment, each serving simultaneously as a delivery rail and a rapid-pacing platform. This report places the case in the context of the relevant literature, highlights the role of individualized Heart Team planning in increasingly complex structural heart disease, and discusses how this biventricular guidewire strategy may contribute to procedural simplification in selected high-risk patients.

## 2. Case Presentation

A 73-year-old woman was referred to our Structural Heart Disease Program because of progressive exertional dyspnea, markedly reduced exercise tolerance, and recurrent symptoms of chronic heart failure corresponding to NYHA functional class III. Her clinical history reflected the natural evolution of advanced rheumatic multivalvular heart disease over almost two decades and included multiple surgical procedures, progressive degeneration of previously implanted prosthetic valves, permanent cardiac pacing, and ultimately the development of severe symptomatic native AS.

The patient’s cardiovascular history began in 2006, when she underwent open-heart surgery for severe rheumatic mitral stenosis associated with severe functional tricuspid regurgitation (TR) and permanent atrial fibrillation with a slow ventricular response. Surgical treatment consisted of mechanical mitral valve replacement with a 27 mm Sorin Bicarbon prosthesis (Sorin Group, Saluggia, Italy), De Vega tricuspid annuloplasty, and implantation of a single-chamber ventricular pacemaker for persistent bradyarrhythmia. Long-term oral anticoagulation with acenocoumarol was initiated and maintained thereafter, reflecting the combined indication of the mechanical mitral prosthesis and permanent atrial fibrillation.

Over the subsequent eight years, the patient remained clinically stable. In 2014, she was hospitalized for exertional dyspnea; echocardiography demonstrated a normally functioning mitral prosthesis, preserved LV systolic function, and moderate tricuspid regurgitation.

In February 2018, routine device follow-up identified an elective replacement indicator for the pacemaker generator after approximately twelve years of continuous service, given that the patient was pacemaker-dependent. Generator replacement was performed uneventfully, with preservation of the original ventricular lead implanted in 2006. Echocardiography performed during the same admission again confirmed normal mechanical mitral prosthetic function, preserved LV systolic performance, and moderate TR; no tricuspid intervention was considered necessary at that stage.

Clinical deterioration became evident during the first half of 2019, with progressive exertional dyspnea, orthopnea, peripheral edema, and repeated episodes of right-sided heart failure requiring hospitalization. Echocardiographic assessment demonstrated marked progression of TR to severe, with annular dilatation and right-sided volume overload. Degenerative changes in the native aortic valve were also becoming apparent, although AS remained only moderate and the symptoms were considered secondary to the dominant tricuspid pathology. Intensive intravenous diuretic therapy produced marked clinical improvement, with resolution of congestion and a weight reduction of approximately seven kilograms. According to contemporary echocardiographic recommendations, TR severity should be assessed using an integrative multiparametric approach, with further subclassification of severe TR into severe, massive, and torrential grades when adequate quantitative data are available. However, the historical 2019 dataset did not contain all variables required for reliable retrospective subclassification beyond severe.

Despite temporary stabilization, recurrent decompensation occurred a few months later, prompting readmission with symptomatic right heart failure and comprehensive preoperative evaluation. Coronary angiography excluded obstructive coronary artery disease, and right-heart catheterization confirmed the hemodynamic consequences of severe TR. After multidisciplinary institutional discussion, repeat surgery was considered the most appropriate option, since medical therapy alone could no longer control symptoms or prevent further RV deterioration. Following dental sanitation and optimization of anticoagulation, the patient underwent redo cardiac surgery in December 2019, consisting of repeat median sternotomy with replacement of the native tricuspid valve by a 31 mm Edwards Perimount bioprosthesis (Edwards Lifesciences, Irvine, CA, USA). The postoperative course was uneventful, with complete elimination of severe TR, satisfactory prosthetic function, and subsequent reverse remodeling of the RV.

The underlying valvular disease nevertheless continued to evolve. Progressive calcific degeneration of the native aortic valve became increasingly evident, while the mechanical mitral prosthesis, which had functioned normally for almost two decades, gradually developed fibrous pannus formation along the posterior aspect of the sewing ring. Although mitral obstruction produced no immediate indication for intervention, these findings illustrated the cumulative structural burden imposed by long-standing rheumatic heart disease and previous valve surgery.

**Figure 1:**
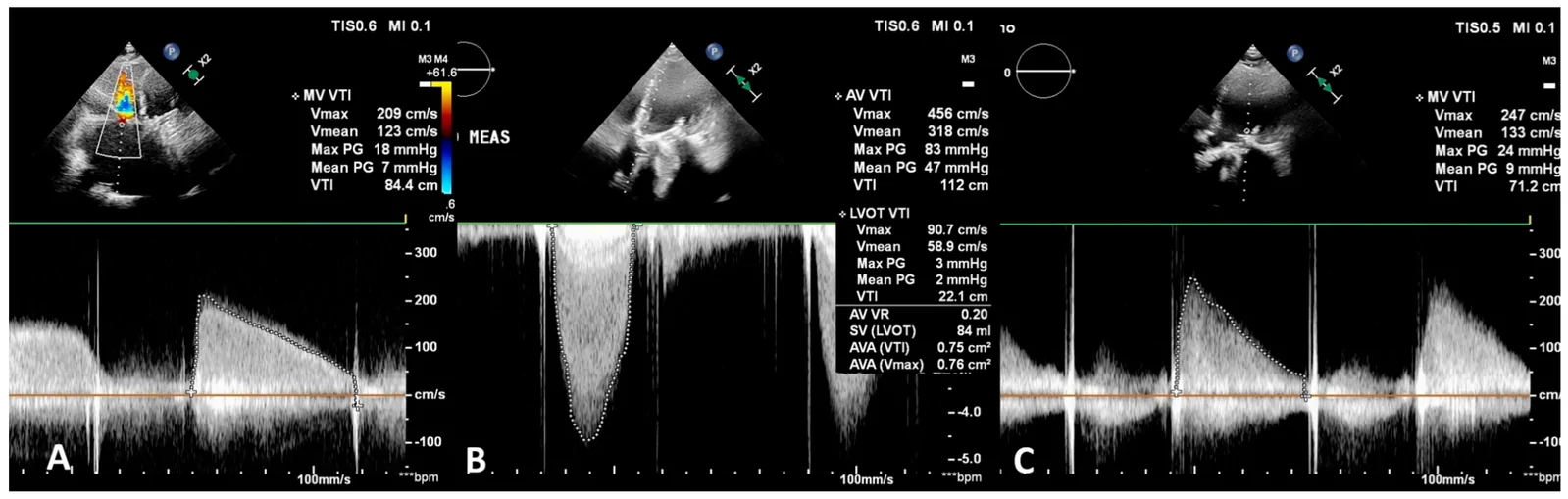
Preprocedural transthoracic echocardiography (apical views). (**A**) Continuous-wave Doppler across the tricuspid bioprosthesis, showing a mean gradient of 7 mmHg (peak velocity 2.1 m/s), consistent with severe prosthetic stenosis. (**B**) Continuous-wave Doppler across the native aortic valve, showing a peak velocity of 4.56 m/s and a mean gradient of 47 mmHg; pulsed-wave Doppler of the left ventricular outflow tract (LVOT) is shown alongside for continuity-equation calculation of an aortic valve area of 0.75–0.76 cm^2^, consistent with critical aortic stenosis. (**C**) Continuous-wave Doppler across the mechanical mitral prosthesis, showing a mean gradient of 9 mmHg, consistent with moderate prosthetic stenosis. *** bpm = beats per minute.

In May 2026, the patient presented with progressive limitations of physical activity, exertional dyspnea on minimal effort, and recurrent symptoms of chronic heart failure despite optimized medical therapy. Comprehensive multimodality imaging was undertaken to define the mechanism of clinical deterioration and guide further management. Transthoracic echocardiography demonstrated severe calcific AS, with a maximal transvalvular velocity of 4.56 m/s, a mean pressure gradient of 47 mmHg, and an estimated aortic valve area of 0.75–0.76 cm^2^ by the continuity equation (Figure 1B), consistent with critical hemodynamic obstruction. LV systolic function had declined compared with previous examinations, with an ejection fraction of approximately 50%, and concentric LV hypertrophy had become more pronounced, reflecting chronic pressure overload.

Transesophageal echocardiography demonstrated pannus formation involving approximately one-quarter of the circumference of the mechanical mitral prosthesis, resulting in moderate prosthetic stenosis (mean gradient 9 mmHg; Figure 1C) with only mild intraprosthetic regurgitation; the severity of obstruction was considered insufficient to justify immediate intervention. The most striking additional finding concerned the surgically implanted tricuspid bioprosthesis, which, despite satisfactory early postoperative performance, showed clear evidence of structural valve degeneration after approximately six years, with marked leaflet thickening and restricted opening producing severe prosthetic tricuspid stenosis (mean gradient 7 mmHg, peak velocity 2.1 m/s; Figure 1A) associated with moderate transprosthetic regurgitation.

**Figure 2:**
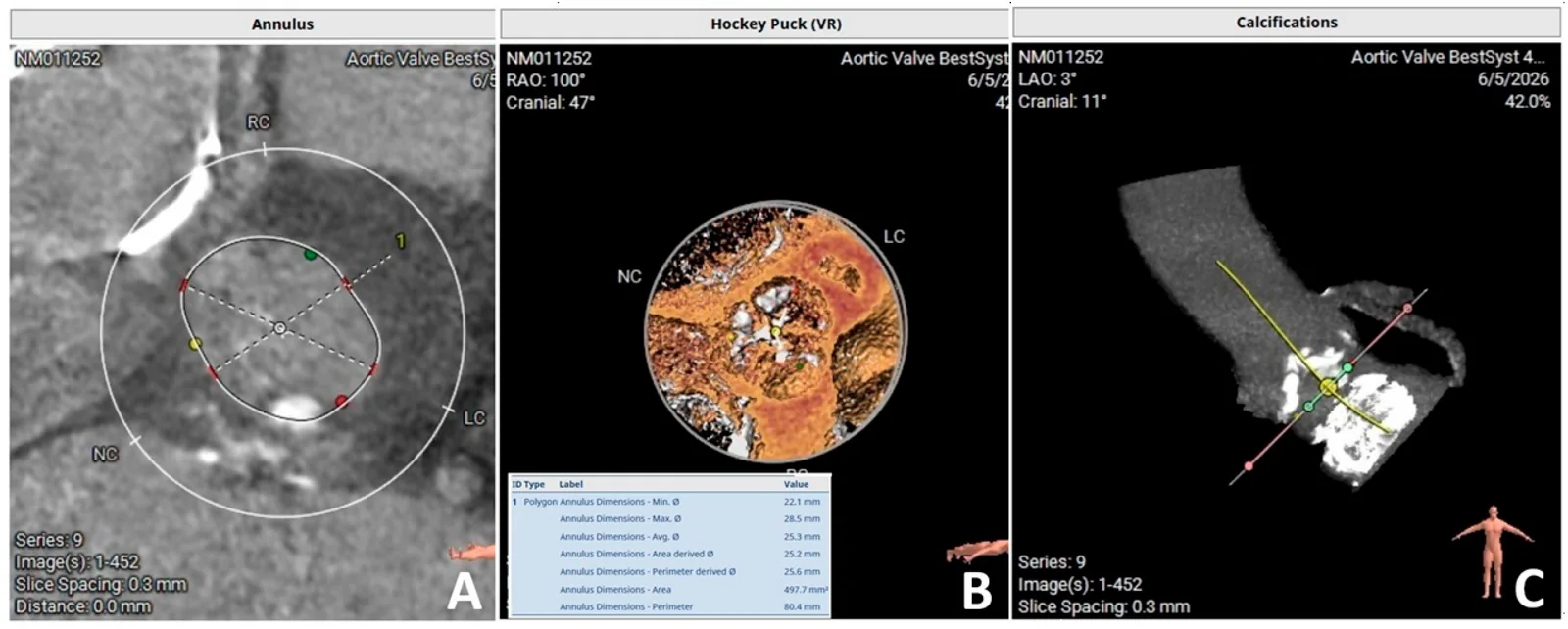
Preprocedural cardiac computed tomography—multiplanar reconstruction used for sizing and calcium assessment of the native aortic valve. (**A**) Annular measurements: minimum diameter 22.1 mm, maximum diameter 28.5 mm, average diameter 25.3 mm, area-derived diameter 25.2 mm, perimeter-derived diameter 25.6 mm, area 497.7 mm^2^, perimeter 80.4 mm. (**B**) Volume-rendered short-axis (“hockey-puck”) view of the aortic root at the level of the valve, showing the non-coronary (NC) and left coronary (LC) cusps. (**C**) Three-dimensional quantification of aortic valve calcification.

Computed tomography (CT) confirmed favorable iliofemoral vascular access for transfemoral intervention and provided detailed anatomical assessment of both the native aortic annulus (Figure 2) and the failed tricuspid bioprosthesis. Coronary angiography again excluded significant epicardial coronary artery disease, confirming that the patient’s symptoms were almost entirely attributable to progressive multivalvular dysfunction rather than to ischemic heart disease.

The case was subsequently discussed by the institutional Heart Team, comprising interventional cardiologists, cardiac surgeons, multimodality imaging specialists, cardiac anesthesiologists, and electrophysiologists. A third open-heart operation, involving replacement during the same operation of the native aortic valve and the failed tricuspid bioprosthesis with possible intervention on the mechanical mitral prosthesis, was judged technically feasible but associated with prohibitively high operative risk given the previous multiple sternotomies, advanced age, pulmonary hypertension, chronic atrial fibrillation, a permanent pacing lead, obesity, and multiple comorbidities (estimated EuroSCORE II of 27.37% and an STS PROM for isolated aortic valve replacement predicted operative mortality of 5.42%, alongside a 14% risk of major morbidity or mortality) [3]. Conversely, the anatomical characteristics of both the native aortic valve and the surgical tricuspid prosthesis were considered suitable for transcatheter treatment. A staged transcatheter approach was discussed but ultimately considered less attractive, since it would require repeated vascular access, repeated anesthesia, and additional manipulation of intracardiac devices. After careful multidisciplinary evaluation, the Heart Team elected to perform a single-stage transcatheter intervention, consisting of transfemoral balloon-expandable transcatheter aortic valve implantation (TAVI) followed immediately by tricuspid valve-in-valve implantation (TViV) during the same procedural session. This strategy required meticulous procedural planning, particularly regarding guidewire positioning, rapid ventricular pacing, and management of the chronic trans-tricuspid pacing lead in this pacemaker-dependent patient, all of which became central determinants of procedural success.

## 3. Procedural Planning and Intervention

Following multidisciplinary discussion, the procedure was scheduled as a single-stage transfemoral intervention under general anesthesia with continuous transesophageal echocardiographic and fluoroscopic guidance. The primary objectives were relief of the critical native AS and treatment of the severely degenerated tricuspid bioprosthesis, while minimizing procedural complexity and avoiding unnecessary manipulation of the chronic transvenous pacing lead crossing the tricuspid valve.

**Figure 3:**
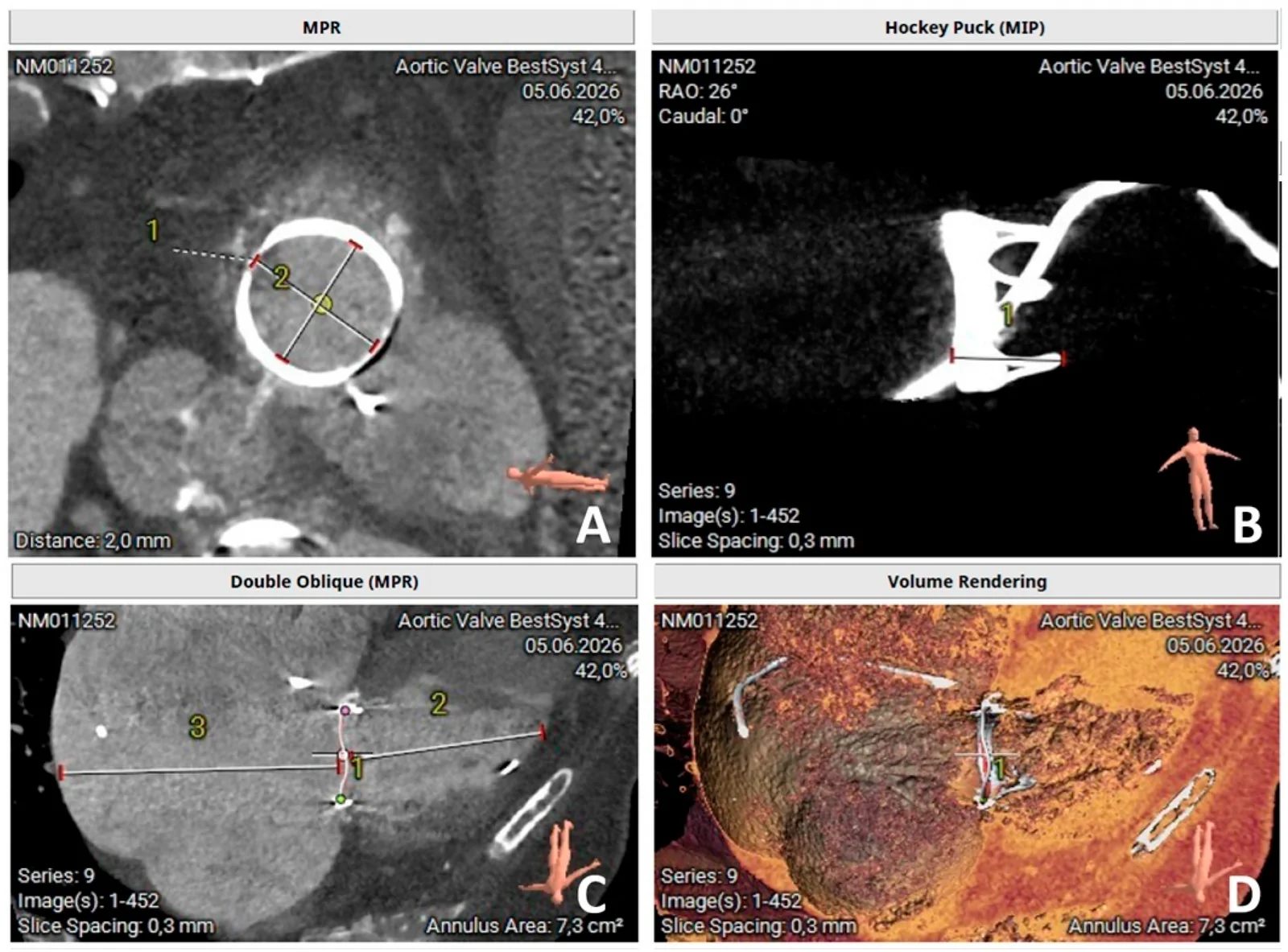
Tricuspid bioprosthesis sizing and its spatial relationship with the pacing lead. (**A**) Axial CT reconstruction showing the tricuspid bioprosthetic sewing ring and its spatial relationship with the ventricular pacing lead. (**B**) Multiplanar CT reconstruction illustrating the relationship between the tricuspid bioprosthesis and the ventricular pacing lead. (**C**) Double-oblique multiplanar reconstruction used for anatomical measurements, showing a tricuspid valve annulus area of 7.3 cm^2^; distance 2, measured from the sewing ring to the right ventricular apex, was 63.1 mm, and distance 3, measured from the tricuspid valve sewing ring to the roof of the right atrium, was 91.2 mm. (**D**) Volume-rendered reconstruction illustrating the three-dimensional spatial relationship between the tricuspid bioprosthesis and the ventricular pacing lead.

Preprocedural cardiac CT demonstrated favorable iliofemoral vascular anatomy for transfemoral access and confirmed appropriate aortic annular dimensions for balloon-expandable valve implantation (average annular diameter 25.3 mm, perimeter 80.4 mm, area 497.7 mm^2^; Figure 2A), together with extensive aortic valve calcification (Figure 2B,C); the deteriorated tricuspid bioprosthesis showed suitable internal dimensions for TViV treatment with a balloon-expandable prosthesis (Figure 3). Fluoroscopic projections and CT images were carefully reviewed before the intervention to define the optimal implantation angles for both valves and to evaluate the spatial relationship between the tricuspid prosthesis and the permanent pacing lead.

Because the patient presented with critical native AS, treatment of the aortic valve was intentionally planned as the first procedural step, since relieving the LV outflow obstruction before addressing the right-sided lesion was expected to improve immediate hemodynamic stability and reduce the risk of circulatory compromise during the remainder of the intervention.

An additional procedural challenge was posed by the permanent ventricular pacing lead, implanted two decades earlier. Advancing additional catheters or stiff guidewires through the RV carries a recognized risk of lead displacement, entrapment, insulation damage, or conductor fracture, particularly during manipulation within a failed bioprosthesis [9]. For this reason, every effort was made to minimize unnecessary exchanges and right-sided instrumentation throughout the intervention. Notably, the presence of a ventricular electrode, even when located outside the bioprosthetic sewing ring, can interfere with the distal portion of the delivery system balloon during inflation. This potential interaction favors the alternative approach of deploying the stiff wire within the RV, which minimizes interference by ensuring better baseline coaxiality from the onset of deployment. Furthermore, since this mechanical interaction may compromise ventricular electrode stimulation effectiveness, maintaining a temporary pacemaker on standby is essential.

To avoid placing two large 14 Fr sheaths on the same side, we used both the right femoral vein and the left femoral artery instead. After obtaining bilateral access with standard percutaneous techniques, systemic anticoagulation was achieved with intravenous unfractionated heparin to maintain an activated clotting time above 250 s. A guidewire was positioned in the LV apex after crossing the severely calcified native aortic valve. We used the INNOWI TAVI wire (Symedrix GmbH, Regensburg, Germany) dedicated pre-shaped 0.035-inch stainless steel guidewire featuring a PTFE coating and 3-Zone Technology designed for transcatheter aortic valve implantation. This guidewire provided stable support for transcatheter aortic valve implantation and simultaneously served as the platform for LV pacing during valve deployment, a strategy validated in a randomized comparison with conventional RV temporary pacing, which found equivalent procedural success and safety while eliminating the need for a separate transvenous pacing lead in most patients [10].

**Figure 4:**
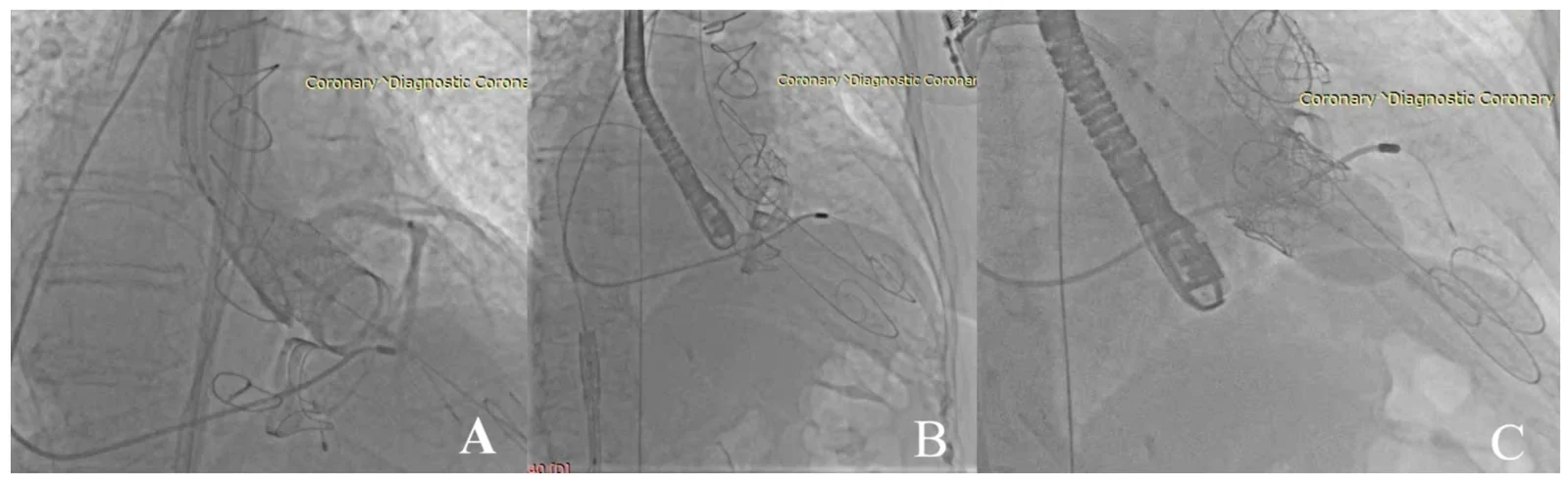
Procedural fluoroscopic imaging. (**A**) Balloon inflation under rapid ventricular pacing during transcatheter aortic valve implantation. (**B**) Simultaneous fluoroscopic visualization of both INNOWI guidewires positioned in the left and right ventricles (biventricular guidewire strategy). (**C**) Balloon expansion of the reverse-mounted tricuspid valve-in-valve prosthesis.

TAVI was performed first, using a balloon-expandable Edwards Sapien 3 Ultra 26 mm prosthesis (Edwards Life Sciences, Irvine, CA, USA) appropriately sized according to preprocedural CT measurements, at nominal pressure. Rapid ventricular pacing was delivered through the LV INNOWI guidewire during valve deployment, providing excellent device stability despite severe annular calcification (Figure 4A). Fluoroscopic and echocardiographic assessment immediately after implantation demonstrated optimal prosthesis expansion, correct positioning, complete anchoring within the native annulus, and the absence of significant paravalvular regurgitation, with hemodynamic measurements confirming complete relief of the critical transaortic obstruction.

A second INNOWI guidewire was subsequently advanced across the degenerated tricuspid bioprosthesis and carefully positioned within the RV under continuous fluoroscopic monitoring, with particular attention to avoiding interaction with the chronic pacing lead. Stable positioning of both guidewires before valve implantation (Figure 4B) allowed intervention to be performed without repeated ventricular catheterization or additional guidewire exchanges. For stiff wire deployment, positioning within the pulmonic trunk is generally preferred to maximize procedural stability. Although this approach may introduce a degree of uncoaxiality compared to RV placement, a slow balloon inflation strategy allows the system to gradually achieve optimal coaxial alignment. Ultimately, we selected RV positioning using a pre-shaped wire with a sufficiently large radius, an approach strongly recommended to ensure adequate system stabilization along the superior-inferior axis.

The old valve was predilated with a 23/40 mm balloon. Edwards Sapien 3 balloon-expandable valve (Edwards Lifesciences, Irvine, CA, USA) was mounted in the reverse orientation required for implantation in the tricuspid position and advanced into the failed surgical bioprosthesis, an approach consistent with the technique used in the largest published series of transcatheter TViV implantation [5,6]. Particular attention was paid to maintaining coaxial alignment with the surgical valve frame while preserving unrestricted mobility of the chronic pacing lead. Edwards Sapien 3 Ultra 29 mm valve (Edwards Lifesciences, Irvine, CA, USA) deployment at nominal pressure was performed under fluoroscopic and transesophageal echocardiographic guidance. Effective rapid ventricular pacing may occasionally be beneficial to enhance stability during deployment. The INNOWI guidewire was maintained in the LV for backup pacing in case of permanent lead damage, though acute pacing was not required as the permanent lead remained functional in this pacemaker-dependent patient. The prosthesis expanded symmetrically within the surgical valve ring (Figure 4C) and achieved stable anchoring without evidence of migration or embolization. Careful fluoroscopic inspection throughout the procedure demonstrated preserved integrity of the permanent pacing lead.

**Figure 5:**
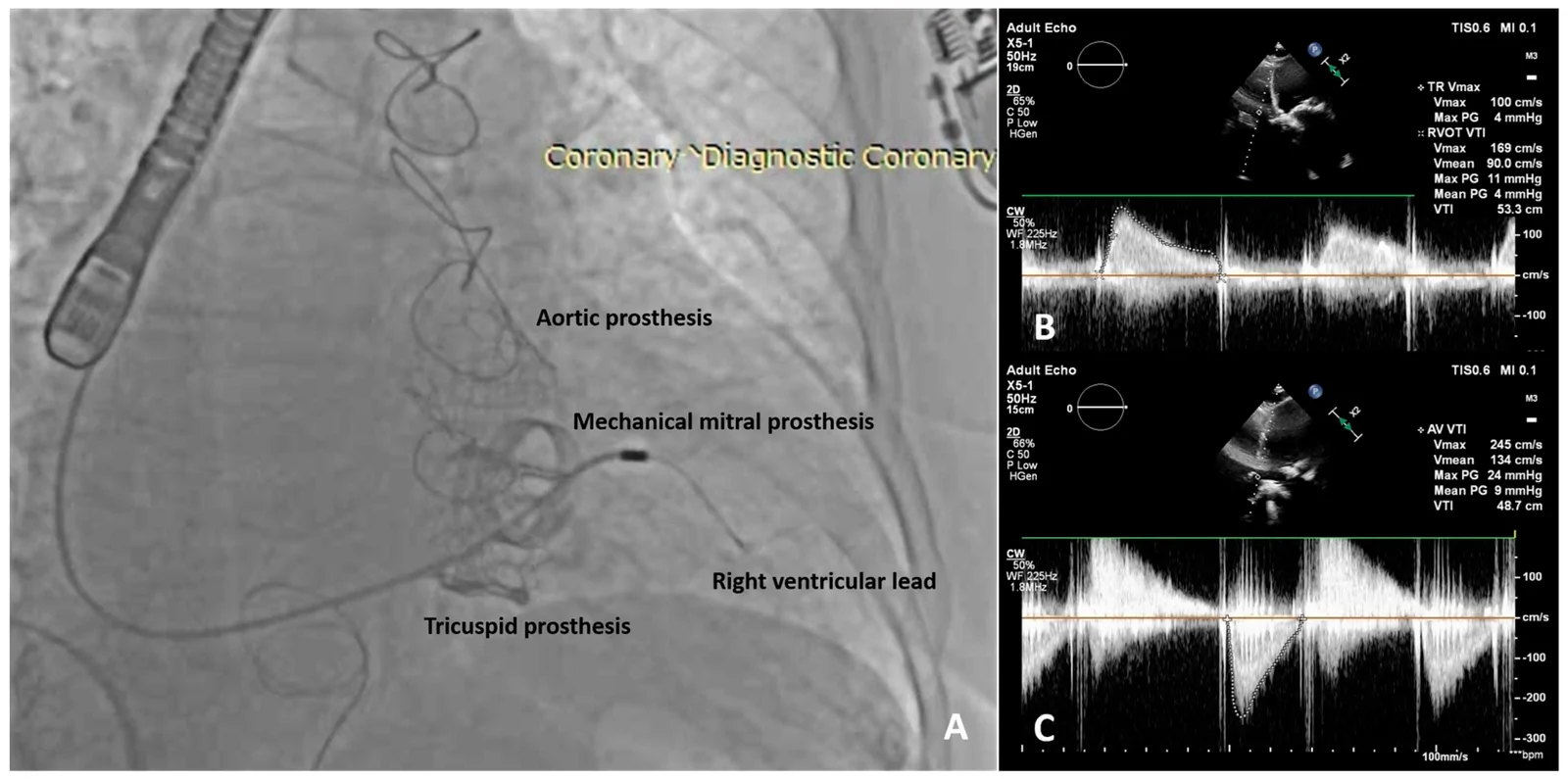
Final procedural and echocardiographic results. (**A**) Fluoroscopic image showing the three prosthetic valves (aortic, mechanical mitral, and tricuspid) together with the permanent right ventricular pacing lead. (**B**) Pre-discharge transthoracic echocardiography, apical four-chamber view, with continuous-wave Doppler across the tricuspid valve-in-valve prosthesis, showing a markedly reduced mean gradient of 4 mmHg and trivial residual regurgitation compared with the severe pre-procedural stenosis in Figure 1A. (**C**) Pre-discharge transthoracic echocardiography, apical five-chamber view, with continuous-wave Doppler across the transcatheter aortic valve, showing a mean gradient of 9 mmHg compared with the critical pre-procedural stenosis in Figure 1B. *** bpm = beats per minute.

Final echocardiography demonstrated excellent function of both newly implanted prostheses. The transcatheter aortic valve showed normal leaflet motion with minimal transvalvular gradients and no significant paravalvular leak, while the TViV prosthesis exhibited unrestricted leaflet mobility, a marked reduction in transvalvular gradient, and only trivial residual regurgitation. Mechanical mitral prosthetic function remained unchanged compared with the preprocedural assessment. Final fluoroscopy confirmed satisfactory positioning of all three prosthetic valves (Figure 5A), and interrogation of the permanent pacemaker demonstrated unchanged sensing thresholds, pacing thresholds, and lead impedance, excluding acute lead dysfunction after the intervention.

The patient was extubated uneventfully and transferred to the cardiac intensive care unit in a stable hemodynamic condition. No conduction disturbances, vascular complications, device embolization, prosthetic dysfunction, or periprocedural neurological events were observed. Pre-discharge transthoracic echocardiography confirmed these findings: the transcatheter aortic valve showed a peak velocity of 2.45 m/s and a mean gradient of 9 mmHg (Figure 5C), consistent with resolution of the pre-procedural critical AS, and the TViV prosthesis showed a markedly reduced mean gradient of 4 mmHg with only trivial residual regurgitation (peak velocity 1.0 m/s, peak gradient 4 mmHg; Figure 5B), consistent with resolution of the pre-procedural severe tricuspid stenosis.

## 4. Contemporary Evidence on Single-Session Double-Valve Structural Intervention

Combined, single-session transcatheter treatment of two structurally diseased valves is no longer a technical curiosity, but it remains far from standardized. This section places the present case within the small but expanding literature on single-session double-valve transcatheter intervention, and develops from that literature a practical framework covering patient selection, procedural sequencing, device choice, and—given the specific challenge posed by this case—pacing strategy and management of pre-existing pacing hardware.

### 4.1. Patient Selection

Patient selection for single-session transcatheter treatment of two diseased valves builds on, but does not simply add together, the criteria developed for isolated TAVI and isolated transcatheter tricuspid valve intervention (TTVI). Both lesions must independently justify treatment, their combination must justify a single-session strategy rather than a staged one, and the presence of any pre-existing intracardiac hardware must be factored into the overall risk assessment from the outset.

Who benefits? The most defensible candidates are patients in whom two valve lesions are each hemodynamically significant and symptomatic, in whom conventional or redo surgery carries prohibitive risk, and in whom both target valves are anatomically suitable for transcatheter treatment. Prohibitive surgical risk in this population is most often driven by multiple previous sternotomies: contemporary reoperative valve surgery series report operative mortality rising from approximately 1.4% for elective procedures to 8% for urgent and over 37% for emergency reoperations, and from roughly 1.6% in NYHA functional class I to more than 20% in class IV [3]. Each additional sternotomy compounds this risk through mediastinal adhesions, prolonged cardiopulmonary bypass, and a higher probability of injury to previously implanted prostheses or grafts during re-entry. When, as in the present case, a patient has already undergone two prior median sternotomies for mitral and tricuspid surgery, a third operation to treat newly critical native AS together with a failed tricuspid bioprosthesis is frequently judged prohibitive, and a combined transcatheter strategy becomes the more attractive alternative.

Anatomical suitability must be established independently for each valve. Aortic candidacy follows standard TAVI criteria (annular dimensions, coronary height, calcification pattern, vascular access). Candidacy for TViV or valve-in-ring treatment depends on the internal dimensions of the failed bioprosthesis or ring, an intact sewing structure capable of anchoring a balloon-expandable frame, and freedom from pannus or thrombus severe enough to compromise deployment; the internal dimensions of the failed prosthesis are also a key determinant of the orifice area achievable after valve-in-valve implantation [5]. When both anatomies are favorable and vascular access is adequate for the additional catheters required by a combined procedure, a single-session strategy becomes a reasonable option for Heart Team discussion.

**Contraindications.** Beyond the standard contraindications to TAVI or TTVI individually—active endocarditis, intracardiac thrombus, inadequate vascular access without a viable alternative route, or very limited life expectancy from non-cardiac disease—the 2025 ESC/EACTS guidelines highlight a caveat of particular relevance to combined procedures: in patients with severe RV dysfunction or precapillary pulmonary hypertension, tricuspid intervention risks futility, and optimal medical therapy is preferred over TTVI [4]. This threshold matters even more in a combined session than in isolated TTVI, since adding a technically successful TViV procedure to a hemodynamically futile substrate exposes the patient to additional procedural time and risk without a realistic prospect of benefit [11].

Registry data nonetheless suggest that RV dysfunction and pulmonary hypertension should be treated as risk-stratifying variables rather than blanket contraindications: in a TriValve registry subanalysis of 300 patients with RV dysfunction, pulmonary hypertension, or both, procedural success remained high (80.7%), and midterm mortality was more closely associated with renal function and hepatic congestion than with the RV–pulmonary hemodynamic abnormality itself [12]. In practice, severe, fixed precapillary pulmonary hypertension or advanced RV–pulmonary artery uncoupling should prompt caution about adding a tricuspid procedure to the same session, favoring either a staged approach or aortic treatment alone with close surveillance of the tricuspid lesion.

**The Heart Team**. Multidisciplinary evaluation is a guideline-level requirement for complex valvular disease in general, and its scope must expand for combined double-valve transcatheter procedures. Beyond the standard core of interventional cardiologists, cardiac surgeons, and multimodality imaging specialists, cases in which a chronic transvenous pacing lead crosses the target tricuspid prosthesis—as in the present report—require early, structured involvement of an electrophysiologist, both to plan lead protection during the procedure and to anticipate contingency pacing needs [9].

Anesthesiology input is equally important, given the longer combined procedure time and the hemodynamic transitions between the two interventions. In the present case, multidisciplinary consensus explicitly weighed the technical feasibility of a third sternotomy against the anatomical suitability of both valves for transcatheter treatment, and concluded that a single-session transcatheter strategy offered the most favorable balance of risk and benefit for this specific patient.

Surgical risk stratification. A persistent limitation in this field is that the most widely used surgical risk scores were derived for single-valve, first-time operations and only partially translate to combined, reoperative, transcatheter scenarios. The Society of Thoracic Surgeons Predicted Risk of Mortality (STS-PROM) and EuroSCORE II remain the most familiar general cardiac surgical risk tools but are known to underperform for isolated tricuspid disease. The dedicated TRI-SCORE, derived from 466 patients undergoing isolated tricuspid valve surgery and incorporating age, NYHA class, signs of right heart failure, diuretic dose, renal function, bilirubin, and LV function, better reflects the risk profile of tricuspid disease and has since been examined across surgical and transcatheter tricuspid cohorts alike [13]. None of the available scores, however, has been validated specifically for combined, same-session aortic and tricuspid transcatheter procedures, and multidisciplinary consensus currently relies on composite clinical judgment that integrates conventional surgical risk, tricuspid-specific risk stratification, frailty, and the anatomical feasibility of a single-session strategy. The patient described here—advanced age, permanent atrial fibrillation, two previous sternotomies, obesity, pulmonary hypertension, and a permanent pacing system—illustrates the type of multi-domain risk profile that no single available score fully captures, reinforcing the case for individualized, multidisciplinary decision-making over reliance on any single instrument in this population [3,4].

### 4.2. Why Single-Session Instead of Staged?

Once a patient has been judged suitable for transcatheter treatment of both valves, the Heart Team faces a second, largely independent decision: whether to treat both lesions in the same session or to stage them across two separate procedures.

**Advantages of a single-session strategy**. A single-session approach exposes the patient to one anesthetic, one vascular access procedure, and one hospital admission rather than two, which is relevant in the elderly, multimorbid patients who typically present with this combination of disease. When a chronic transvenous pacing lead crosses the tricuspid valve, as in the present case, minimizing the total number of right-heart instrumentations is itself a safety argument, since each additional crossing of the tricuspid apparatus with catheters, sheaths, or guidewires adds a discrete, cumulative risk to the lead [9]. A combined procedure also corrects left- and right-sided hemodynamics within the same physiological window, potentially producing a more complete improvement in forward cardiac output than isolated correction of either lesion, and avoids the interval during which an untreated second lesion continues to impose its hemodynamic burden.

**Disadvantages**. Combining two procedures lengthens anesthetic and fluoroscopy time and increases cumulative contrast exposure in a population in which renal function is frequently impaired. A complication during the first valve implantation may compromise the safety margin available for the second, and a single session affords less opportunity to reassess the patient clinically and hemodynamically between interventions than a staged strategy does. Vascular and venous access planning is also more demanding, since large-bore arterial and venous access must be secured and managed within one setting.

What the literature shows. Evidence for simultaneous double-valve transcatheter treatment remains observational and is drawn mainly from case reports and small series, including the two most directly comparable prior reports [7,8] and reports involving other multivalvular combinations [14,15,16,17,18]. No randomized trial or dedicated registry has simultaneously compared staged double-valve transcatheter strategies, and no specific guideline currently defines the preferred approach [15,19]. The choice therefore remains individualized.

**Critical commentary**. In the absence of comparative data, a pragmatic framework can nonetheless be proposed. A single-session strategy is most defensible when (i) both valves are anatomically straightforward, with a predictable combined procedure time; (ii) baseline hemodynamic reserve is adequate to tolerate two sequential interventions without an interval for recovery; (iii) chronic intracardiac hardware makes repeated instrumentation specifically undesirable, as here; and (iv) institutional resources—operator experience, procedure-room time, anesthesia support—allow the combined procedure without compromising either individual step. A staged strategy should be favored when either valve’s anatomy is borderline and may require unplanned adjuncts (e.g., bioprosthetic valve fracture, alternative access), when baseline instability favors a shorter individual procedure, or when institutional experience with the specific combination is limited. In the present case, favorable anatomy for both valves, adequate hemodynamic reserve, and the specific imperative to avoid repeated right-heart instrumentation around the chronic pacing lead together supported the Heart Team’s decision to proceed with a single-session strategy.

### 4.3. Which Valve First?

When both valves are treated in the same session, a further decision—largely unaddressed as an explicit topic in the literature—is the sequence in which they should be treated. The physiological reasoning differs meaningfully depending on which valves are involved and, critically, on whether the non-aortic lesion is a fixed structural obstruction or a functional, potentially reversible one.

**AS + MR.** When AS coexists with secondary functional MR, MR may improve after isolated TAVI as relief of afterload promotes favorable LV remodeling [20,21]. This potential reversibility supports an aortic-first, staged strategy with reassessment of the mitral lesion, reserving simultaneous treatment mainly for MR unlikely to improve, such as primary/degenerative MR [17].

**AS + TR**. The present case illustrates a materially different scenario. Unlike functional MR, which arises largely from LV remodeling that TAVI can reverse, the tricuspid lesion here was structural degeneration of a surgically implanted bioprosthesis: a fixed, mechanical obstruction that cannot be expected to improve with afterload reduction alone. This distinction matters directly for sequencing: when the tricuspid lesion is a degenerated bioprosthesis or annuloplasty ring, rather than functional native TR secondary to annular dilation or pulmonary hypertension, deferring its treatment in anticipation of spontaneous improvement is not physiologically sound, which strengthens the argument for treating both lesions in the same session rather than staging. Functional native TR behaves more like functional MR in this respect: cohort data show that moderate-to-severe TR also frequently regresses after isolated TAVI—from 32.9% to 15% of patients in the same cohort cited above [20]—although baseline TR severity remains independently associated with prognosis [21], and a meaningful minority of patients show persistence or progression rather than regression.

Once the decision to treat both valves in the same session has been made, sequencing should generally follow the dominant hemodynamic driver. Treating critical AS first, as in the present case, relieves LV outflow obstruction and stabilizes systemic pressure before the right heart is instrumented; because rapid ventricular pacing is required for balloon-expandable deployment on both sides, performing the more hemodynamically destabilizing step against a fixed aortic obstruction while ventricular filling and right-sided function are still intact is generally preferable to performing it after the right heart has already been manipulated.

Ventricular interdependence means that acutely relieving one-sided obstruction can alter loading conditions on the opposite ventricle. Significant right-sided disease may also reduce transvalvular flow and mask the true severity of low-flow, low-gradient AS, complicating diagnosis and sequencing decisions.

**Toward an algorithm**. Valve order can reasonably be organized around two questions: (1) is the non-aortic lesion structural/fixed or functional/potentially reversible, and (2) which valve is the dominant driver of current hemodynamic instability? A structural, non-reversible lesion—a degenerated bioprosthesis, as here—generally argues for treating both valves in the same session regardless of order, since no benefit accrues from staging in anticipation of an improvement that will not occur. A functional, potentially reversible lesion generally argues for staging: treating the aortic valve first and reassessing. Within a same-session strategy, AS should generally be treated first unless a dominant, hemodynamically destabilizing tricuspid lesion argues for the reverse. This logic forms the second decision node of the practical algorithm proposed in Section 4.8.

### 4.4. Procedural Planning

Successful execution of a combined double-valve transcatheter procedure depends on preprocedural planning that treats both valves as a single integrated system rather than as two independent targets.

**Computed tomography**. A single full-cycle, ECG-gated acquisition can serve both valves. For the aortic component, standard multiplanar reconstruction provides annular measurements, coronary height, and calcification burden, as performed in the present case (Figure 2). The same dataset can be reconstructed to profile the tricuspid bioprosthesis or annuloplasty ring—its stent frame geometry, degree of pannus or calcification, and, critically, its spatial relationship to any pre-existing pacing lead—information essential for anticipating whether guidewire or catheter manipulation is likely to contact the lead during deployment. CT also defines iliofemoral vascular access suitability for the large-bore sheaths required by balloon-expandable delivery systems, and in complex or borderline anatomy can be used to construct patient-specific 3D-printed models to rehearse device delivery before the actual procedure, an approach reported in complex patients undergoing sequential aortic and tricuspid transcatheter treatment [22].

**Transesophageal echocardiography**. Transesophageal echocardiography provides real-time intraprocedural guidance for both steps: continuous visualization of guidewire position relative to native structures and the pacing lead, monitoring of device deployment, and immediate assessment of paravalvular regurgitation after each valve is implanted, complementing fluoroscopy throughout the combined procedure.

**Sizing**. Aortic sizing follows standard TAVI algorithms based on annular dimensions derived from CT. TViV sizing follows a fundamentally different logic: rather than measuring a native annulus, the operator must determine the true internal diameter—as opposed to the manufacturer’s labeled size—of the failed bioprosthesis, since labeled size can overestimate true internal diameter by 1 to 4 mm depending on stent and leaflet design [23]. A dedicated, freely available sizing application, built around this “true internal diameter” concept, catalogs the true internal diameter and recommended transcatheter valve size for essentially all commercially available surgical bioprostheses and rings, and has become a standard preprocedural tool for valve-in-valve planning in any position [23].

**Fluoroscopic views**. Aortic deployment uses a coplanar projection aligning the three aortic cusps, typically predefined from CT. TViV deployment requires distinct projections, since the tricuspid annulus lies in a different spatial plane from the aortic root; adequate en face visualization of the failed bioprosthesis or ring typically requires separate fluoroscopic angles from those used for the aortic valve. Both optimal angle sets should ideally be predefined from preprocedural CT rather than sought empirically during the procedure, to limit contrast use and fluoroscopy time in what is already a longer combined intervention.

**Vascular access.** As in the present case, transfemoral arterial access for the aortic valve and transfemoral venous access for the TViV can typically be obtained from the same or contralateral groins, avoiding additional access sites. When transfemoral venous access is unsuitable—for example, because of occlusion or a hostile inferior vena cava—alternative routes including transjugular and bilateral cervical approaches have been described for complex combined transcatheter procedures, illustrating that access planning, like device planning, must be individualized to each patient’s anatomy rather than assumed from a default transfemoral strategy [18,24].

### 4.5. Choice of Prosthesis

**Balloon-expandable platforms**. For valve-in-valve treatment of a failed tricuspid bioprosthesis or ring, balloon-expandable platforms—as used for both valves in the present case—remain the dominant, most extensively reported option, reflecting both the largest available registry experience [5] and practical advantages specific to this anatomy: precise, operator-controlled positioning during a brief period of rapid pacing, and, because the existing surgical sewing ring or annuloplasty band provides the anchoring structure, no requirement for the device’s own frame to achieve independent annular fixation. The same balloon-expandable platform used for the aortic valve can therefore be reused, in reversed orientation, for the TViV step, preserving procedural familiarity with a single delivery system across both interventions, as described in the largest published TViV series [5,6].

**Reverse mounting**. Balloon-expandable transcatheter valves are designed and manufactured for anterograde deployment in the aortic position. Implantation in the tricuspid position, approached from the inferior vena cava, requires the valve to be manually remounted onto its delivery system in reversed orientation before the procedure, so that the leaflets open in the correct direction once deployed within the failed surgical valve. This reverse-mounting technique, now standard for tricuspid and mitral valve-in-valve procedures using balloon-expandable platforms, does not require different sizing logic from standard mounting, but does require operators specifically trained in, and familiar with, the modified preparation sequence.

**Self-expanding platforms.** Self-expanding valves are established in aortic intervention, with broadly comparable clinical outcomes to balloon-expandable devices in native AS [25], but they are used less frequently for tricuspid valve-in-valve treatment. Most self-expanding tricuspid experience involves dedicated devices for native TTVR with anchoring mechanisms distinct from the surgical sewing-ring fixation used in the present TViV case [26].

**Sizing principles.** Regardless of platform, valve-in-valve sizing balances two competing risks: undersizing, which risks device migration, embolization, or paravalvular leak, against oversizing, which risks incomplete leaflet expansion and elevated residual gradients. Preprocedural determination of the true internal diameter of the failed prosthesis, rather than reliance on its labeled size, is the foundation of this balance for any platform choice [23], and was the approach followed in the present case for both the aortic annulus and the degenerated tricuspid bioprosthesis.

### 4.6. Pacing Strategies

Among all elements of procedural planning for combined double-valve transcatheter treatment, pacing strategy merits the most extended discussion, both because rapid ventricular pacing is mechanically required for balloon-expandable valve deployment at both the aortic and tricuspid positions, and because pre-existing intracardiac pacing hardware, as in the present case, transforms what is often a routine procedural step into a central determinant of overall strategy (Table 1).

**Conventional RV pacing**. The traditional approach to rapid pacing during TAVI, and the default approach used for the tricuspid step in most published TViV series, is placement of a dedicated temporary pacing wire in the RV apex via a separate venous access site. This approach is familiar, reliable, and does not depend on the valve delivery guidewire remaining in a specific position. Its principal disadvantages in the present context are the need for an additional venous puncture beyond those already required for the procedure itself, and—most importantly when a chronic pacing lead already crosses the tricuspid valve—an additional instrumentation of the right heart with a second wire that must itself avoid interaction with both the existing lead and, during the tricuspid step, the TViV delivery system.

**LV guidewire pacing**. For the aortic component of a combined procedure, pacing through the stiff guidewire used for valve delivery has become an established alternative to a separate temporary wire. In the randomized EASY TAVI trial, LV pacing via the delivery guidewire achieved procedural success and safety comparable to conventional RV temporary pacing, while reducing procedure duration, fluoroscopy time, and the need for additional venous access in most patients [10]. Capture occurs because the distal guidewire tip, positioned in direct contact with the LV endocardium, functions as a unipolar pacing cathode when connected via a sterile adaptor to an external pacing generator, with a surface electrode or the arterial sheath itself serving as the return anode; because a guidewire tip provides a larger, less stable contact surface than a dedicated bipolar pacing electrode, effective capture typically requires higher current outputs than conventional temporary pacing. Reported limitations include occasional capture-threshold instability requiring guidewire repositioning—particularly during the mechanical excursion associated with balloon inflation or valve deployment—and the need for careful electrical isolation of other equipment in the field to avoid interference with the pacing circuit. Notably, the investigators of that trial explicitly anticipated that the same principle might extend usefully to transcatheter mitral and tricuspid interventions, in which a conventional temporary pacing lead crossing the tricuspid valve can itself interfere with device delivery [10], an anticipation that the biventricular strategy described below extends directly.

**Dedicated pacing guidewires**. Distinct from off-label pacing through a standard delivery-support guidewire, purpose-built pacing guidewires have also been developed and reported in early clinical experience: a dedicated temporary pacing guidewire designed specifically to combine the mechanical support function of a standard stiff guidewire with a reliable pacing electrode at its distal tip, obviating the need for a separate temporary wire without relying on off-label use of a wire not engineered for pacing [27]. Right-sided guidewire pacing during isolated TViV implantation has similarly been reported using a dedicated pacing guidewire advanced into the RV, independent of the delivery system for the valve itself [27]. These dedicated devices address the main theoretical limitation of off-label delivery-wire pacing—that the wire was not engineered with pacing performance as a primary design goal—but remain less widely available and less extensively reported than the off-label technique used in the present case.

Permanent pacemaker considerations and conduction disturbance risk. Conduction disturbance is not a hypothetical concern in this population: it is among the most frequent complications of transcatheter tricuspid valve replacement, reflecting the close anatomical relationship between the tricuspid annulus, the membranous septum, and the atrioventricular conduction axis. In the multicenter TRIPLACE registry, high-grade atrioventricular block occurred in 13.5% of patients within one month of orthotopic TTVR, with 88% of episodes occurring within the first week [29]. A single-center mechanistic analysis reported new-onset conduction disturbance of any type in 44.3% of patients, with new permanent pacemaker implantation required in 14% within 30 days, and identified reduced RV free-wall strain and specific leaflet morphology as predictors [28]. These figures are broadly comparable to, and in some series exceed, the conduction disturbance rates well described after TAVI itself, reported at roughly 3% to 26% depending on valve generation and platform. A dedicated multidisciplinary consensus document has recently been published specifically to address atrioventricular conduction risk in transcatheter tricuspid intervention, underscoring how central this issue has become to the field [30]. For a combined double-valve procedure, this means conduction risk must be anticipated from both the aortic and the tricuspid steps, and that any patient without a pre-existing permanent pacemaker undergoing combined TAVI and TViV treatment should be regarded, from a rhythm perspective, as being at meaningfully elevated risk of requiring permanent pacing from either intervention. In the present, already pacemaker-dependent patient, this risk had already been realized two decades earlier; the procedural task was therefore not to anticipate a new pacing indication but to protect the existing system, discussed further in Section 4.7.

**Leadless pacing**. Leadless pacemakers, implanted entirely within the RV without any transvalvular lead, have emerged as a particularly relevant option for patients with, or at risk of needing, permanent pacing in the context of tricuspid valve disease or intervention, since they avoid the central problem this case addresses: a lead crossing the tricuspid apparatus. The growing case-report literature now describes leadless pacemaker implantation specifically through or across bioprosthetic and transcatheter tricuspid valves, including standard single-chamber devices advanced through a previously implanted bioprosthetic tricuspid valve using dedicated fluoroscopic projections to ensure atraumatic crossing [31], first-in-human combined implantation of a transcatheter tricuspid valve together with a leadless VDD pacemaker providing atrioventricular synchrony [32]. Twelve-month follow-up data indicate that leadless pacemakers do not meaningfully impair atrioventricular valve function [33], and a dedicated “heart team” framework for lead-management decisions—including when to favor leadless over transvenous pacing—in patients undergoing percutaneous tricuspid intervention has been proposed [34]. For patients undergoing combined TAVI and TViV treatment who do not yet have a pacing system, leadless pacing represents an increasingly attractive default should permanent pacing become necessary, since it sidesteps the lead-jailing risk discussed in Section 4.7 entirely; its principal current limitations are the historical restriction to single-chamber pacing (with dual-chamber leadless systems still maturing), and the need for operators experienced in navigating the delivery system across a bioprosthetic or transcatheter valve rather than a native annulus.

Conduction system pacing has been shown feasible after transcatheter tricuspid valve replacement and may preserve physiological ventricular activation while avoiding conventional RV apical pacing, although experience in the combined double-valve setting remains limited [35].

**Backup pacing**: the biventricular guidewire strategy of the present case. The present case’s principal technical contribution lies precisely at the intersection of the strategies above. Rather than choosing between a conventional temporary wire and a dedicated pacing guidewire, dedicated stiff guidewires were positioned in both ventricles before either valve was deployed: one advanced across the native aortic valve into the LV apex, used for active rapid pacing during aortic valve deployment following the established principle of delivery-wire pacing [10]; and a second advanced across the degenerated tricuspid bioprosthesis into the RV, positioned before TViV deployment.

Because the existing permanent pacemaker remained functional throughout, with capture confirmed by device interrogation both before and after the procedure, active pacing through the RV guidewire was not required; instead, it was maintained specifically as a contingency platform, available for immediate use had manipulation around the chronic lead compromised its function. This use of a guidewire as a backup pacing platform, rather than as the primary source of rapid pacing, appears to be a distinct refinement not previously described in this combination: the same off-the-shelf guidewire already required for delivery support served, without modification or additional hardware, as insurance against acute loss of the patient’s only functioning pacing system, while the LV guidewire simultaneously provided active pacing for the aortic step. This biventricular approach—one wire for active pacing on the left, one wire for delivery support and contingency pacing on the right—allowed the procedure to proceed without a separate temporary pacing wire, without additional venous access, and without any deliberate attempt to test or challenge the existing permanent system beyond what was necessary for valve deployment.

**Synthesis: choosing a pacing strategy.** The choice among these strategies can be organized around two patient-specific questions: does the patient already have a functioning permanent pacing system, and if so, does a transvalvular lead cross the valve about to be treated? A patient with no pre-existing pacing system and low anticipated conduction risk is reasonably managed with delivery-wire pacing on both sides, as established for the aortic position [10] and, by extension, for the tricuspid position when a dedicated pacing guidewire or an off-label support wire is used with appropriate caution [27]. A patient with no pre-existing system but substantial anticipated conduction risk—extensive calcification near the conduction axis, pre-existing bundle branch block, or a TRIPLACE-type high-risk profile [29,30]—may be better served by primary implantation of a leadless pacemaker as part of the same or a closely staged admission. A patient with an existing transvenous system whose lead does not cross the valve being treated requires standard precautions but faces comparatively low incremental risk. A patient with an existing transvenous system whose lead does cross the target valve, as in the present case, benefits most from the combination described here: careful preprocedural imaging of the lead–prosthesis relationship (Section 4.4), a strategy that minimizes additional right-heart instrumentation (Section 4.2), guidewire-based contingency pacing rather than a separate temporary wire, and—for future, comparable patients in whom permanent pacing has not yet been established—early consideration of leadless pacing to avoid recreating this problem altogether.

### 4.7. Management of Permanent Pacing Leads

When a chronic transvenous pacing or defibrillator lead crosses the tricuspid annulus, TViV or valve replacement confronts the electrophysiology and structural teams with a genuine dilemma, recently and aptly described in the literature as the choice between leaving the lead “jailed” or “freed” [36]. The present case illustrates one resolution of this dilemma—careful positioning that avoided jailing altogether—but a comprehensive review must also address the scenarios in which jailing is unavoidable or has already occurred.

**Lead jailing.** Jailing leaves a pre-existing transvalvular lead trapped between the new valve frame and the native annulus or surgical prosthesis. Although early valve-in-valve reports suggested that jailed leads could remain functional [37,38], more recent reports and cohorts have documented clinically relevant lead abnormalities and complications after transcatheter tricuspid valve replacement [36,39,40]. Mechanical impingement by the valve frame appears to be an important mechanism. Current expert consensus therefore favors avoiding jailing, particularly in pacemaker-dependent patients, patients with previously therapeutic defibrillator leads, multiple transvalvular leads, relevant infection risk, or high lead tension, while acknowledging that the evidence remains observational [41].

**Extraction.** When extraction is chosen—either electively before valve deployment or later, if a jailed lead fails or becomes infected—it carries risks distinct from routine transvenous lead extraction. In the largest general lead-extraction registry, procedure-related major complications occur in a small but consequential minority of all transvenous extractions, and risk rises with lead dwell time [42], which is typically prolonged in this population (a median of nearly nine years in one contemporary tricuspid cohort [40]). Extracting a lead that is already jailed by a transcatheter or surgical valve frame is more demanding still: because a laser or powered sheath cannot safely be advanced past the valve frame without risking damage to the prosthesis or cardiac perforation, the distal, jailed portion of the lead can typically only be freed using traction with a locking stylet rather than mechanical sheath advancement, and technique reports describe adjunctive maneuvers such as femoral snaring to provide counter-traction when a standard superior approach alone is insufficient [43]. Older expert guidance treated a lead trapped by a prior tricuspid valve replacement as an effective contraindication to later extraction; more recent case reports demonstrate that extraction of a jailed lead is feasible in expert hands using these adjunctive techniques, but at meaningfully higher procedural complexity and risk than extraction of a free-lying lead, reinforcing that the decision to jail a lead at the time of the index valve-in-valve procedure should be made with the awareness that it may be difficult to reverse [43].

**Repositioning.** An intermediate strategy—repositioning the existing lead before valve deployment, rather than either extracting it entirely or leaving it in its original trajectory—is feasible in some patients and can reduce the degree of mechanical interaction between lead and valve frame without the risks of full extraction. This requires favorable lead redundancy and venous anatomy, close preprocedural imaging of the lead’s course relative to the planned valve landing zone, and, as with extraction, careful preprocedural transesophageal echocardiographic assessment for lead-associated vegetation or thrombus, since manipulation of an infected or thrombus-laden lead carries embolic risk. Repositioning does not eliminate lead–valve interaction as completely as extraction or a lead-independent pacing strategy would, but avoids the specific mechanical challenges of extracting a lead through a valve frame that has not yet been implanted.

**Long-term implications**. Whatever strategy is chosen, chronic mechanical interaction between a transvalvular lead and a transcatheter valve frame warrants structured long-term surveillance rather than a single postprocedural check. Device interrogation—assessing pacing and sensing thresholds and lead impedance—should be performed immediately after the procedure, as in the present case, and repeated at scheduled intervals thereafter, since dysfunction in jailed leads has been shown to emerge progressively over months to years rather than exclusively at the time of implantation [36,40]. For pacemaker-dependent patients with an old or already-marginal lead, some authors now advocate considering a more deliberately protective strategy—for example, converting to a leadless system, or extracting and repositioning the lead, before an elective valve-in-valve procedure, rather than defaulting to jailing and monitoring for failure after the fact—although this remains an individualized, Heart Team-level decision rather than a codified recommendation [36,41]. In the present case, meticulous preprocedural imaging of the lead’s course and careful intraprocedural positioning of the valve-in-valve prosthesis relative to the existing lead avoided jailing altogether, illustrating that prevention, where anatomically feasible, remains preferable to managing a jailed lead after the fact.

### 4.8. Proposed Practical Algorithm

**Figure 6:**
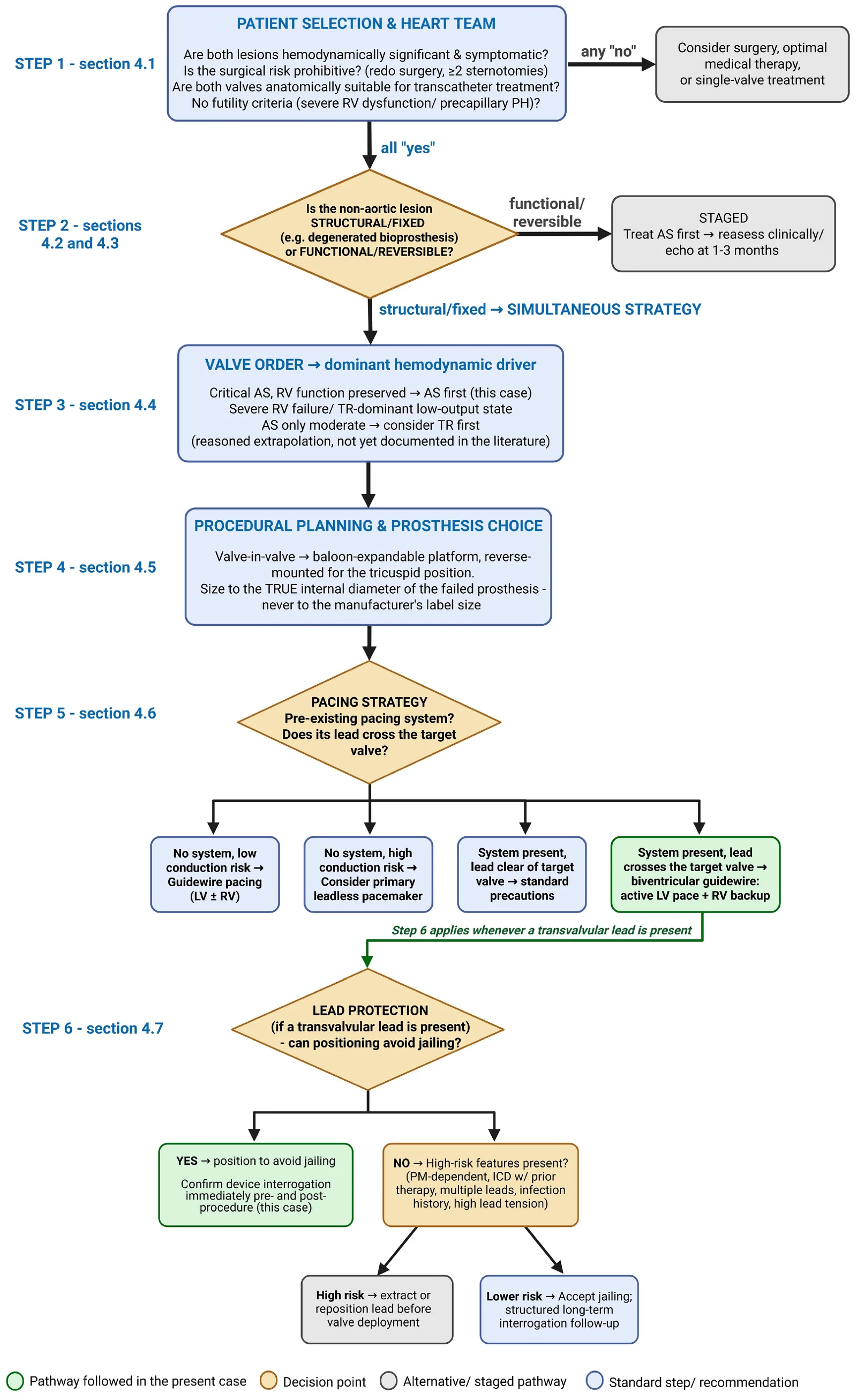
Proposed practical algorithm for combined transcatheter aortic and tricuspid valve-in-valve intervention. Green boxes indicate the pathway followed in the present case; amber diamonds indicate decision points; grey boxes indicate alternative or staged pathways; blue boxes indicate standard steps or recommendations. Section numbers in parentheses indicate where the supporting evidence for each step is discussed in the text.

The preceding sections can be synthesized into a single practical algorithm (Figure 6), organized around six sequential decisions a multidisciplinary consensus must make when a patient presents with both critical AS and a failed tricuspid bioprosthesis or ring: (1) whether the patient is a reasonable candidate for transcatheter treatment of both valves at all; (2) whether treatment should be single-session or staged, based principally on whether the non-aortic lesion is structural or functional; (3) which valve to treat first when a single-session strategy is chosen; (4) which prosthesis and sizing strategy to use; (5) which pacing strategy best fits the patient’s existing hardware and anticipated conduction risk; and (6) how to protect any pre-existing transvalvular pacing lead.

The algorithm is deliberately organized as a series of binary or categorical decisions rather than a rigid protocol, reflecting the genuinely individualized, multidisciplinary, discussion-dependent nature of these decisions in a field where, as emphasized throughout this review, high-quality comparative evidence remains scarce.

The pathway followed in the present case—highlighted in green in Figure 6—required a same-session strategy driven by a structural tricuspid lesion, an AS-first sequence, balloon-expandable valves in both positions, a biventricular guidewire strategy providing active LV pacing and RV contingency pacing, and careful positioning that avoided jailing the existing permanent pacing lead altogether.

We propose this algorithm not as a definitive protocol, but as a structured starting point for Heart Team discussion that can be refined as larger, prospective, multicenter data on combined double-valve transcatheter intervention become available.

### 4.9. Limitations

An important limitation of the present report is the relatively short duration of follow-up, reflecting the recent timing of the intervention. Consequently, the current report focuses on procedural feasibility, immediate hemodynamic results, and early clinical outcome; longer-term clinical, echocardiographic, prosthetic-valve, and pacing-system surveillance will be necessary to assess the durability of these findings.

## 5. Conclusions

This case of single-stage transcatheter treatment for critical native AS and a degenerated tricuspid bioprosthesis, in a patient with a mechanical mitral prosthesis and a chronic trans-tricuspid pacing lead, illustrates both the technical feasibility and the procedural complexity of combined double-valve structural intervention. Maintaining dedicated guidewires in both ventricles throughout the procedure provided pacing and delivery support without additional venous access or instrumentation of the existing pacing lead, allowing both valves to be treated safely within a single session.

More broadly, this review highlights that single-session double-valve transcatheter intervention remains an evidence-poor field, supported almost entirely by case reports and small series rather than randomized or registry-level comparative data. Within this limited evidence base, several practical principles nonetheless emerge: patient selection should weigh anatomical suitability, surgical risk, and futility criteria for both valves jointly rather than independently; the distinction between structural, fixed lesions and functional, potentially reversible ones should guide the choice between single-session and staged treatment; the hemodynamically dominant lesion should generally be treated first; and pacing strategy and lead protection deserve at least as much preprocedural planning as valve sizing itself, particularly as leadless and conduction-system pacing options continue to mature. The practical algorithm proposed in Section 4.8 synthesizes these principles into a structured starting point for Heart Team discussion, to be refined as larger, prospective, multicenter experience with combined double-valve transcatheter intervention accumulates.

The favorable early result in this case underscores the importance of multimodality imaging, individualized Heart Team decision-making, deliberate valve sequencing, a predefined pacing strategy, and protection of pre-existing intracardiac hardware when planning complex multivalvular transcatheter intervention.

## Figures and Tables

**Table 1:** Comparison of pacing strategies available during combined transcatheter aortic and tricuspid valve procedures.

Strategy	Extra Venous Access	Extra Dedicated Hardware	Risk to an Existing Transvalvular Lead	Best Suited to
Conventional RV temporary wire	Yes	No	Adds a further instrumentation of the right heart	Any patient; default when guidewire pacing is unavailable or unreliable
LV guidewire pacing (aortic step)	No	No	Not applicable (left-sided)	The aortic component of any TAVI, combined or isolated [10]
Dedicated pacing guidewire	No	Yes (purpose-built wire)	Minimal, if used as the sole RV wire	Operators wanting reliable pacing performance without off-label use [27]
New permanent pacemaker	Depends on approach	Yes (full system)	Not applicable (no pre-existing lead)	Patients with substantial anticipated conduction risk and no existing system [28,29,30]
Leadless pacemaker	No (delivered via femoral vein)	Yes (full system)	None (creates no transvalvular lead)	Patients with, or at risk of needing, permanent pacing where a transvalvular lead is specifically undesirable [31,32,33,34]
Guidewire as contingency/backup (present case)	No	No	None beyond standard valve deployment	Pacemaker-dependent patients with an existing, functional transvalvular lead crossing the target valve

## Data Availability

The original contributions presented in this study are included in the article. Further inquiries can be directed to the corresponding author.

## Abbreviations

The following abbreviations are used in this manuscript:

AS	aortic stenosis
CIED	cardiac implantable electronic device
CT	computed tomography
EACTS	European Association of Cardio-Thoracic Surgery
ESC	European Society of Cardiology
LV	left ventricle
MR	mitral regurgitation
NYHA	New York Heart Association
PTFE	Polytetrafluoroethylene
RV	right ventricle
STS-PROM	Society of Thoracic Surgeons Predicted Risk of Mortality
TAVI	Transcatheter aortic valve implantation
TR	tricuspid regurgitation
TRIPLACE	Transcatheter Tricuspid Valve Replacement Registry
TTVI/TTVR	transcatheter tricuspid valve intervention/replacement
